# Development of Phage Lysins as Novel Therapeutics: A Historical Perspective

**DOI:** 10.3390/v10060310

**Published:** 2018-06-07

**Authors:** Vincent A. Fischetti

**Affiliations:** Laboratory of Bacterial Pathogenesis and Immunology, The Rockefeller University, 1230 York Avenue, New York, NY 10065, USA; vaf@rockefeller.edu

**Keywords:** phage, lysins, endolysins, lytic enzymes, discovery, therapeutic, antibiotic resistance, alternative to antibiotics

## Abstract

Bacteriophage lysins and related bacteriolytic enzymes are now considered among the top antibiotic alternatives for solving the mounting resistance problem. Over the past 17 years, lysins have been widely developed against Gram-positive and recently Gram-negative pathogens, and successfully tested in a variety of animal models to demonstrate their efficacy. A lysin (CF-301) directed to methicillin resistant *Staphylococcus aureus* (MRSA) has effectively completed phase 1 human clinical trials, showing safety in this novel therapeutic class. To validate efficacy, CF-301 is currently the first lysin to enter phase 2 human trials to treat hospitalized patients with MRSA bacteremia or endocarditis. If successful, it could be the defining moment leading to the acceptance of lysins as an alternative to small molecule antibiotics. This article is a detailed account of events leading to the first therapeutic use and ultimate development of phage-encoded lysins as novel anti-infectives.

## 1. Preface

Since 2001, when our first paper was published describing the in vivo use of phage enzymes as potential therapeutics, I have been asked many times: “How did you think of doing this?” This article is a detailed answer to that question. I certainly was not the only person in the world working on phage lysins at the time; however, I was one of the few working with a highly-active lysin against Gram-positive bacteria. It was clearly a constellation of several factors, the most important of which were my background and being in the right place at the right time.

## 2. Phages

Bacterial viruses, or bacteriophages, are the most abundant microbial agent on Earth, with an estimated 10^31^ particles, ten times more than their bacterial counterparts. Because of their vast numbers, phages permeate the entire biosphere from the soil to marine environments [1], the atmosphere [2] and the human body [3]. Estimates suggest that >10^10^ phages transcytose across the gut into our tissues each day [3]. Through their perpetual lytic proliferative cycle, phages maintain the balance of all ecosystems on the planet (even those in the human body) by modulating the composition of indigenous bacterial communities. Because of this, and the lack of apparent predators, there is little doubt that phages control the biosphere, since they sit atop the biological food chain.

## 3. Lysis from Without

Lysis of bacteria can occur through the addition of an outside agent resulting in what is termed “lysis from without”. This lytic (L) event can occur either through the exposure of the bacteria to a high multiplicity of infection of phage (L_P_) [4,5] or the exposure of the bacteria to phage-derived or other wall-degrading lysins (L_L_) [6]. For several reasons, it is likely that these events do not occur in nature to any great extent, if at all, but are a consequence of phage or lysin manipulation in the laboratory. Both events utilize enzymes that cleave the peptidoglycan; in the case of L_P_, lytic enzymes are part of the phage tail structure used to locally degrade the peptidoglycan of either Gram-positive or Gram-negative bacteria, to allow entry of phage DNA into the cell. L_L_ occurs if lysins used by the phage to release progeny happen to interact with sensitive bacteria. In general, this lysis from without is more likely to occur with Gram-positive bacteria since their cell wall peptidoglycan is more directly exposed on the cell surface than Gram-negative bacteria, which is protected by an outer membrane. Because both lytic events are rapid and efficient, the power of these enzymes could be harnessed to control bacterial pathogens.

## 4. Early Lysin Discovery

Phage lysins are highly evolved cell wall hydrolytic enzymes used to rupture phage-infected bacteria and release progeny phage particles into the environment. Since lysins lack leader sequences, they are synthesized and sequestered within the cytoplasm of the cell along with assembling phage particles. Lysin molecules from phage that infect Gram-positive bacteria are two-domain structures that reach their peptidoglycan substrates in a coordinated event with the holin, a phage-encoded molecule that accumulates in patches within the bacterial membrane, ultimately forming a hole at a time programmed into the structure of the holin molecule [6]. The hole allows the lysin to translocate across the membrane into the peptidoglycan. The high-affinity characteristic of the lysin’s binding domain drives the molecule to its specific peptidoglycan substrate, resulting in cleavage by the catalytic domain. Peptidoglycan bonds neighboring the hole continue to be cleaved, and as a result of the 10 to 15 atmospheres of cytoplasmic pressure, the cytoplasmic membrane externalizes and explodes releasing the progeny phage. It is important to note that lysins from Gram-positive phage closely resemble fungal cellulases [7,8], which are similarly constructed enzymes with both cell wall-binding and catalytic domains joined by a flexible linker. It is unknown whether lysins, like cellulases, use their binding domains to bind and slide across the wall peptidoglycan as they cleave [7] or are simply enzymes that stay substrate-bound and are only able to cleave adjacent bonds, requiring several molecules for a lytic event. Nevertheless, it is believed that their high-affinity binding may have evolved to prevent the diffusion of free lysin molecules after lysis, resulting in the unintentional killing of adjacent (potential) host bacteria. It is estimated that half of the bacteria on Earth are killed through the lytic cycle every 48 h, making lysins the most effective and widespread bactericidal agent on the planet [9].

Nearly all lysins from DNA phage of Gram-positive bacteria have modular structures defined by N-terminal catalytic domains and C-terminal cell wall-binding domains [10,11,12]. Phage lysins primarily from staphylococci and mycobacteria have also evolved multiple catalytic domains with different specificities [13]. A major exception to this structural motif is the C1 phage lysin (described below). This 114-kDa lysin is comprised of three diverse proteins; the binding domain consists of eight 8 kDa subunits that form a doughnut interacting with two catalytic domains with different specificities (an amidase and a glycosidase). Significantly, the C1 lysin is 200 times more active in its cleavage activity than any other lysin reported to date [14]. The entire structure of the C1 lysin is completely novel, so its origins remain a mystery.

In the early 1900s, shortly after Twort [15] and d’Herelle [16] described the existence of bacteriophages as agents that kill bacteria, Clark and Clark [17] isolated a phage from a Milwaukee WI sewage treatment plant that they termed “sludge phage”. They learned that this phage was able to propagate in streptococci that infected only animals (Lancefield group C) but not those that infected humans (group A). Evans used the same sludge phage (renamed B563) in her studies showing that lysates of this phage had a lytic activity on strains of streptococci that the phages themselves were unable to infect [18]; she termed the phenomena “nascent lysis” and suggested that the activity may be equivalent to the lysin activity previously reported by Twort [19]. Maxted [20] received the B563 phage from Evans and further characterized the “lytic factor” showing that it: (i) killed groups A, C and E streptococci independent of the presence of active phage; (ii) was active over a pH range of 6.5–8.6; and (iii) was a protein, based on protease sensitivity. He speculated that this “lytic factor” was similar to the lytic substance or lysins reported at the time to be present in Gram-negative phage lysates [20].

Krause, a physician in the laboratory of Maclyn McCarty (of Avery, MacLeod and McCarty [21]) at the Rockefeller University renamed the Evans B563 phage C1 because of its activity on group C streptococci, and was the first to partially purify the C1 lysin. Starting with a fresh crude lysate of the C1 phage on group C streptococci, he purified away most of the phage and major contaminants by a series of ammonium sulfate precipitation and ultracentrifugation steps. The final active solution was clear and stable when stored frozen or in a lyophilized state [22]. In the ensuing years, this C1 phage lysin was a laboratory tool used by Krause and colleagues in the McCarty laboratory at Rockefeller to digest the cell wall of groups A and C streptococci in a series of elegant papers characterizing the wall carbohydrate and protein components of these pathogens [22,23,24,25]. In fact, as a result of these publications, the *S. pyogenes* cell wall was one of the best characterized at the time. The C1 lysin was also a useful enzyme to completely remove the cell wall of group A streptococci in the presence of hypertonic sodium chloride to produce and study viable streptococcal protoplasts or L-forms [26]. While around the same time (1960s), lysins were described in the lysates of *Staphylococcus aureus* (the most common of which was termed virolysin) [27,28], only one group reported the partial purification of a staphylococcal lysin [29,30]. While there were sporadic publications regarding lysins from Gram-positive and Gram-negative bacteria in the 1980s and 1990s, the great majority of the publications occurred after 2000.

Several attempts to further purify the C1 phage lysin to homogeneity met with some success and indicated that enzyme activity was dependent on free sulfhydryl groups, a pH optimum of 6.1, and displayed a molecular weight of >100,000 Da [31], the latter of which we now know is unusual for phage lysins. One of the challenges in purifying this lysin was the loss of activity during the purification process as a result of the irreversible binding of heavy metals and thiol-alkylation of the cysteine in the active site of the cysteine, histidine-dependent amidohydrolase/peptidase (CHAP) domain [32].

Fischetti entered the scene in the 1960s, working as a technical assistant for John Zabriskie, a physician in the McCarty laboratory at the Rockefeller University working on streptococcal-related diseases such as rheumatic fever and scarlet fever. Zabriskie was particularly interested in the scarlet fever toxin of *S. pyogenes* since Barksdale at New York University (30 blocks south of Rockefeller University on the east side of Manhattan) published that the diphtheria toxin was phage-encoded [33]. Could the scarlet fever toxin also be phage encoded? Fischetti was hired to help Zabriskie with his experiments on streptococcal phages while Zabriskie tended to his patients at the Rockefeller Hospital [34]. While working at Rockefeller during the day, Fischetti would drive to Brooklyn in the evening to Long Island University to take courses towards a master’s degree, which he received in 1966. He was then accepted into the PhD program at the New York University School of Medicine where arrangements were made that he would take courses at NYU but do his thesis work in the McCarty laboratory at Rockefeller, under the direction of Bernheimer at NYU and Zabriskie at RU. It was decided that for his thesis Fischetti should purify to homogeneity the C1 phage lysin, since Krause only partially purified the enzyme and a pure lysin would be more useful to perform further cell wall extractions in the laboratory. Thus, for his thesis, Fischetti [35] solved the stability problem of the C1 lysin by using sodium tetrathionate to reversibly block and thus protect the sulfhydryl group in the CHAP domain before purification, then after purification the lysin could be reactivated by releasing the tetrathionate with reducing agents prior to use. In the early 1970s, Fischetti—now a Rockefeller faculty member—used this purified C1 lysin in the ensuing decades as a tool to aid in the extraction and characterization of the streptococcal M protein and reveal the method by which M protein and other surface proteins on Gram-positive bacteria were anchored to the cell wall [36,37,38].

## 5. The Right Person at the Right Time

The information accumulated in the Fischetti laboratory regarding the characteristics of streptococcal M protein—including its α-helical coiled-coil fibrous structure [39] and its complete sequence [40]—guided his laboratory towards the development of a vaccine to prevent streptococcal pharyngeal infection (i.e., strep throat). Beachey, using a pepsin extraction method of whole streptococci, identified the type-specific N-terminal region of the M molecule, which was distal to the cell wall, and antibodies to this region showed type-specific protection in a mouse model [41,42,43]. Fischetti, using his sequence data, discovered a region of the M molecule located proximal to the cell wall that was conserved among all M protein serotypes [44,45]. Using synthetic peptides mimicking this conserved region, he found that the peptides delivered orally to mice (linked to cholera toxin B subunit as a mucosal adjuvant) induced mucosal secretory IgA antibodies (sIgA) that protected the animals from streptococcal pharyngeal colonization across different M serotypes, showing cross-protection between M-types for the first time [46,47]. As a result of the body of work on the streptococcal M protein and Gram-positive surface proteins, in 1990 Fischetti became Professor and Head of the Laboratory of Bacterial Pathogenesis and Immunology, the same laboratory that was started by Maclyn McCarty and later headed by Emil Gotschlich [48,49].

During the 1990s, antibiotic resistance was becoming a serious problem. Vancomycin resistance, prevalent among enterococci, began to be seen in strains of *Staphylococcus aureus* and cases of infection by this species with intermediate-level resistance to vancomycin (VISA) were reported [50,51]. If this trend continued, alternatives to conventional antibiotics would be needed. Periodic discussions about the resistance problem among the scientists within the Fischetti laboratory triggered an idea: Could the *S. pyogenes* colonizing the oral cavity of mice be removed with the C1 phage lysin if delivered orally? Having developed a mouse model of streptococcal pharyngeal colonization for his vaccine studies, Fischetti treated some colonized mice orally with lysin and others (the controls) with saline. After swabbing their throats 24 h later, he found that the lysin-treated animals were essentially decolonized of streptococci while the controls remained heavily colonized. After verifying these results several times, the first paper describing a phage lysin as a therapeutic “Enzybiotic” was published by Nelson et al., in PNAS in 2001 [52]. While this was a topical delivery, it indicated for the first time that lysins were active in an in vivo environment. Jutta Loeffler, a Swiss physician in the Fischetti laboratory, published similar results nine months later, but in this instance the purified lysin Pal from the pneumococcal bacteriophage Dp-1 was used [53]. For these experiments, a pneumococcal nasal colonization model was developed which showed, like with the *S. pyogenes* experiments, that nasal delivery of the Pal lysin decolonized the mice from pneumococci.

## 6. Accelerated Development

The success of these first two experiments lead to two grants from DARPA, the Defense Advance Research Project Agency, an arm of the Department of Defense (DOD) that was well known in the field for funding high-risk, high-reward projects. This funding clearly accelerated the development of lysins as therapeutics. The first grant was directed toward developing lysins against *Bacillus anthracis*, and the second was designed to produce lysins against emerging antibiotic resistant pathogens, particularly *S. aureus*.

**Figure 1:**
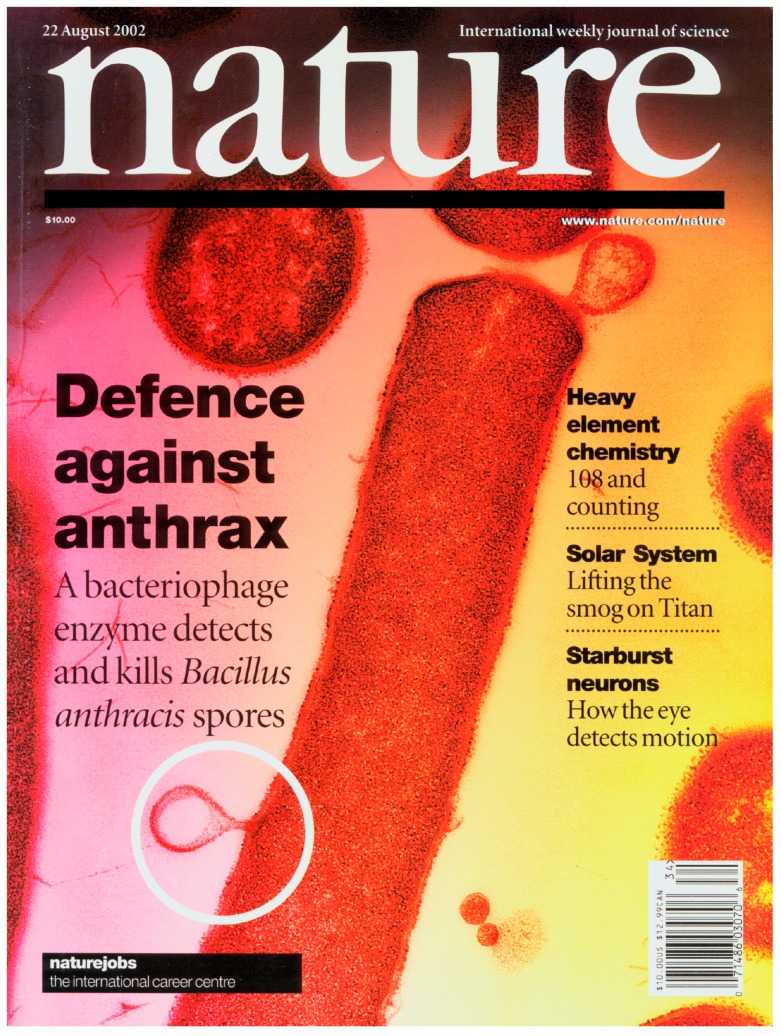
The cover of *Nature*, 22 August 2002, showing the cover article of a phage lysin against *Bacillus anthrax.* The cover image is a bacillus after treatment with the PlyG lysin, showing the cytoplasmic membrane externalizing (white circle and at the top of the bacillus).

For the development of a third therapeutic lysin, the Fischetti laboratory chose a direct approach to identify a lytic enzyme for *B. anthracis.* An expression library of the gamma phage (a phage highly specific for *B. anthracis*) was produced and screened for lytic activity on lawns of live *Bacillus cereus* RSVF1 (a close relative to *B. anthracis*). A lytic clone was identified and the recombinant lysin (called PlyG) was isolated and purified. When used in a mouse model of bacteremia, where the PlyG was delivered intraperitoneally three hours after challenge with a lethal dose of bacilli, the lysin-treated animals were saved from rapid death. This paper was published as the cover article in Nature by Raymond Schuch et al., in 2002, and was the first example of the systemic use for lysins as a therapeutic [54] (Figure 1). The publication date, being about a year after the anthrax letter attack of 18 September 2001, likely motivated the cover since the world was still uneasy about the dissemination of anthrax spores through the mail, and this paper suggested a novel control for this pathogen.

Several novel characteristics of phage lysins were revealed in the next set of publications by Jutta Loeffler et al. [55,56]. In the first, Pal (an amidase) and Cpl-1 (a muramidase), two pneumococcal phage lysins with different peptidoglycan cleavage sites were tested for their in vitro activity, alone and in combination, against several serotypes of *Streptococcus pneumoniae*, including penicillin-resistant strains [55]. The enzymes demonstrated synergism in their ability to cleave the bacterial peptidoglycan and thus in concert may be more efficient for the prevention and elimination of pneumococcal colonization and infection. In a follow-up study [56], the pneumococcal Cpl-1 lysin delivered intravenously to mice that were bacteremic with pneumococci, led to 100% survival at 48 h, compared to 20% survival of buffer-treated controls, verifying the anthrax systemic results. Since lysins are immunogenic proteins, it was anticipated that antibodies produced in response to therapeutic use could render a subsequent lysin treatment ineffective. This was a valid criticism and the Fischetti laboratory was prepared to develop new lysins if this occurred. Fortunately, this was not necessary. Loeffler et al., [56] found that the efficacy of the Cpl-1 lysin to protect mice was not significantly diminished after previous intravenous exposure and antibody production to the lysin. Importantly, no effect on lytic activity was observed even when Cpl-1 was mixed with Cpl-1-specific hyperimmune rabbit serum. Similar results were later repeated in other laboratories with other lysin molecules, suggesting that lysins could be used in situations where multiple administrations were necessary to treat certain disease situations. Finally, this study also showed that the Cpl-1 lysin was specific for all strains of *S. pneumococci* tested but had minimal to no effect on other Gram-positive commensal bacteria, pointing to its species specificity, a characteristic of most lysins. This targeted killing feature of phage lysins is now more accepted, since it diminishes the side effects seen with broad-spectrum antibiotics.

## 7. New Tools

In the early days of lysin development the Fischetti laboratory took several approaches to identify active lysins. Since Rockefeller University is located in Manhattan, next to the East River, one of the best sources of phage was river water. Laboratory members also had excursions to the suburbs to collect soil samples in the woods, compost heaps, and gardens. They were also successful with stool samples from various large animals at a local zoo, as well as sewage and bat guano. Samples were processed to separate the phage from the bacterial population and debris by standard techniques. These viral metagenomes represented a rich source of recombinant proteins. In order to identify the lysin genes, a novel two-step screening technique was developed for cloning the lytic enzymes from the viral DNA. A primary screen was used in which transformed *Escherichia coli* clones that demonstrated lysis on blood agar plates as a result of cloning the holin gene were identified. In a secondary step, the clones identified in the primary screen were overlaid with autoclaved (to permeabilize the outer membrane) gram-negative bacteria as a peptidoglycan source to assay directly for recombinant expression of lytic enzymes, which are often encoded adjacent to holins in phage genomes. This method provided a general approach for identifying lysins from a wide range of uncultured phage [57]. To construct plasmid libraries from small quantities of genomic/metagenomic DNA, a linker amplification technique was developed with topoisomerase cloning which allowed for inducible transcription in *Escherichia coli* [57]. This combination of tools enabled the Fischetti laboratory to identify lysin molecules against a variety of pathogens from which the best was selected for further development.

## 8. Other Laboratories

Not until late 2003 (over two years after the first publication in PNAS) did the first publication about lysin therapy from another laboratory emerge. This was from the Pedro Garcia laboratory in Spain, which verified the results from the Fischetti laboratory using a pneumococcal sepsis model with the same lysins the Fischetti laboratory employed, Cpl-1 and Pal. Since that time, a large number of laboratories around the world have published numerous papers on the identification and use of lysins as therapeutic agents (too many to list here). Because of the independent catalytic and binding domains in the structure of lysins, the Garcia laboratory had been using phage lysins as a model system prior to 2003 to understand the effects of domain swapping on lysin activity and specificity [53,58,59]. With this wealth of information on hand, chimeric lysins were subsequently produced with improved therapeutic characteristics [58,60,61,62].

## 9. An Effective Alternative to Antibiotics

The need for alternatives to antibiotics had been building over the last few decades and had come to a point where decisions needed to be made regarding new therapies to combat antibiotic-resistant bacteria. In a recent detailed review of possible alternatives, Czaplewski et al. examined the therapies that had the best probability of satisfying this need in the shortest time. They concluded, “On the basis of a combination of high clinical impact and high technical feasibility, the approaches anticipated to have the greatest potential to provide alternatives to antibiotics were phage lysins as therapeutics, vaccines as prophylactics, antibodies as prophylactics, and probiotics as treatments…” [63].

By 2015 two companies—ContraFect and Intron Biotechnology—were the first to be in phase 1 human clinical trials using different lysins against *S. aureus* [63]. Now in 2018, only ContraFect is actively in phase 2, testing the treatment of *S. aureus* bacteremia or endocarditis in hospitalized patients worldwide using their lysin CF-301, developed as PlySs2 in the Fischetti laboratory [64]. This trial is planned to be complete by the end of 2018 and, if successful, could be the turning point for the widespread therapeutic use of lysins.
